# The Impact of Linguistic Form of Labels on Desire for Social Distance in Mental Health

**DOI:** 10.1007/s11469-022-00967-y

**Published:** 2022-12-02

**Authors:** Matt Geoffrey Mallinson, Anastasia Giannakopoulou, Andrew James Clements

**Affiliations:** 1 School of Psychology, University of Bedfordshire, Luton, United Kingdom; 2 Aston Business School, Aston University, Birmingham, United Kingdom

**Keywords:** Person first language, identity first language, mental health, essentialism, labelling, stigma

## Abstract

The American Psychological Association, among other influential bodies, make recommendations on language for describing people with mental health conditions. The present studies test the impact of the recommended language on stigma. In Study 1, participants (n = 294) were asked to complete measures of desire for social distance from individuals given a diagnostic label in either person-first possessive, identity-first noun, or identity-first adjective forms. Familiarity with the diagnoses was considered as a potential influence on the outcome. The 3*2 (linguistic form * experience) factorial design was repeated for three diagnoses - schizophrenia, anorexia and alcoholism. In Study 2, the research was replicated with a sample recruited from the UK population via social media (n = 230). Factorial ANOVA was used for analysis. In contrast with previous literature, the studies found neither an effect of linguistic form (hypothesis 1) nor an interaction effect with familiarity (hypothesis 2). Research on this topic is in an early stage and, above all, it remains important to use language, which shows respect when talking to or about others.

## Introduction

Stigma, a negative social attitude towards a characteristic (American Psychological Association, n.d.), cultivates shame and despair for people experiencing mental health issues and can impact critical areas of life (Hinshaw & Stier, 2008). A desire for social distance refers to an unwillingness to engage socially with those with a stigmatised characteristic. It is a core component of stigma and can affect self-esteem, interpersonal relationships, and life opportunities including housing and employment (Jorm & Oh, 2009; Sickel et al., 2014). Changing the language used to label people with mental health conditions is argued to have potential to reduce stigma and improve quality of life (Jensen et al., 2013). Research is necessary to understand how this can be achieved.

Research has been conducted to empirically investigate the effects of different categories of language and their effect on stigma. Central to the discussion of linguistic choices and stigma are ‘person-first’ and ‘identity-first’ language. Person-first language is that which places emphasis on the individual by referring to them before their condition in a sentence. This often involves possessive phrasing (person-first possessive [PFP] form) which gives the person ownership over the condition. e.g. ‘person with schizophrenia’ (Snow, 2007). Identity-first forms, on the other hand, place emphasis on the condition. Often, the condition is referred to in adjective form (Identity-first adjective [IFA] form) preceding a reference to the individual, e.g. ‘schizophrenic person’ (Gernsbacher, 2017), but identity-first language also includes noun form labels which refer to both the condition and the person simultaneously (identity-first noun [IFN] form), e.g. ‘a schizophrenic’ (American Psychological Association, 2020).

Person-first and identity-first language are both argued as preferable by different campaign groups, activists, and academics. Wright (1991) argued that person-first language, by emphasising the person and separating them from the condition, promotes perception of their individuality. It is promoted as an alternative to identity-first language which is said to cast the person as a victim of their disorder or equate them with it (Snow, 2007). A counterargument posits that identity-first language empowers the person by allowing them to reclaim a stigmatised aspect of their identity (Sinclair, 2013). Reflecting these views, the APA have modified guidelines for academics and professionals. While previously the APA recommended person-first rather than identity-first language (American Psychological Association, 2009), both person-first and identity-first language are now considered to be acceptable (American Psychological Association, 2020).

While the APA’s changes in recommendations are an appropriate response to appeals by concerned bodies, there is relatively little scientific research on the topic. Some of the existing evidence raises concerns that IFN labels have essentialising properties, i.e. defining the ‘core’ of who a person is (Maass et al., 2013; Walton & Banaji, 2004). Furthermore, evidence suggests that essentialism is linked to stigmatisation of people with mental health disorders (Howell et al., 2011). It may therefore be expected that IFN forms increase stigma. However, in contrast with this, Penn & Nowlin-Drummond (2001) failed to demonstrate a difference in stigmatising attitudes between those exposed to IFN or PFP mental health labels. However, it should be noted that the number of participants per condition was small, and therefore it would be beneficial to expand Penn & Nowlin-Drummond’s work to a larger sample.

It has been suggested that the effect of language may be mild, and that a single exposure may have relatively little impact on strong pre-existing beliefs regarding mental health conditions (Boland, Patterson & Witten, 1987; Reynaert & Gelman 2007). A significant impact may be more likely with prolonged, repeated exposure or where the mental health condition is unfamiliar and thus free of pre-existing beliefs (Reynaert & Gelman, 2007). However, it may be difficult to test experimentally the effect of long-term exposure. This theorisation is in an early stage and has limited evidence, but has potential to improve understanding of when and how changes in language can be used to tackle stigma. However, as familiarity of a condition is often expected to reduce stigmatization (Felix & Lynn, 2022), it is important to consider a potential moderation effect of familiarity on the impact of linguistic form.

A study by Cuttler & Ryckman (2019) utilised a larger sample size to investigate a difference between the impacts of PFP (n = 149) and IFA (n = 154) form (adjectives were referred to as ‘nouns’ in their report). Participants were randomly assigned to versions of a vignette which differed only in the form of language used to describe the central character. Participants were asked to rate the character on 10 traits which were chosen as stigmatising by the authors, and these ratings were compared between linguistic forms. As summable psychometric scales were not used, the average rating for each of the 10 traits was compared. This was repeated for 5 vignettes involving different mental health conditions. The authors concluded that significant differences in 21 of the 50 ratings demonstrated that IFA forms were more stigmatising than PFP forms. However, the large number of comparisons creates issues with type 1 error due to the multiple comparisons problem (Miller, 1981). Therefore, this work can be built upon by use of an established measure of stigma with a composite score and controlling for number of analyses.

The present paper contributes to the literature by examining whether an effect of linguistic form on stigma can be seen when using a large sample size and an established measure with a composite score. This adds to a growing body of research which can inform choices of language aimed to destigmatise mental illness. It is hypothesised that there is a difference in desire for social distance based on linguistic form used to refer to a person with a mental health condition (Hypothesis 1). The role of familiarity in shaping linguistic effects is also examined. It is hypothesised that there is an interaction between prior familiarity with a mental health condition, defined as direct experience or mere awareness, and linguistic form on desire for social distance (Hypothesis 2).

## Study 1

### Methods

#### Design

A 3*2 (linguistic form * experience) factorial design, with experimental and quasi-experimental (specifically, ‘ex post facto’) elements, was used to address each of four mental health conditions (alcoholism, schizophrenia, agoraphobia, anorexia). An independent variable of linguistic form was presented with three levels; IFN form, PFP form, and IFA form. Familiarity with conditions was recorded as experience or awareness. Experience was recorded at two levels ‘experience’, consisting of those with experience with the conditions (either through knowing someone with it or through experiencing it personally), and ‘no experience’ which consisted of those without. Awareness was also recorded at two levels, but was later discarded as explained in preliminary analysis. The dependent variable measured was ‘desire for social distance’.

### Participants

Of 460 people who started the study, 294 provided usable data. These were composed of 34 males (11.6%), 258 females (87.8%), and 2 people identifying as other genders. Age, among 290 participants who reported it, ranged from 18 to 60 with a mean of 33.09 (SD = 12.20). Participants’ prior experience with each mental health condition is shown in Table 1 below.

**Table 1:** Number of Participants by Familiarity with Condition

	Inexperienced	ExperienceD
Schizophrenia	171	123
AlcoholISM	94	200
Anorexia	132	162

Participants were recruited by convenience sampling method. Participation was open, online, to any person meeting the requirement of being aged 18 years or older. Recruitment was via university student social media groups and emails, and a university student participant pool.

For the study, participants were randomly assigned to a linguistic form condition using the automated randomization feature in Qualtrics software (Qualtrics, 2019). Basic descriptive statistics for each of the groups are shown in Table 2.

**Table 2:** Demographics by Linguistic Form Group

Linguistic form	N	Age Mean	Age SD	Gender (Male/Female/Other)
ifn	95	32.67	12.08	11 M, 83 F, 1 O
pfp	99	34.95	12.66	12 M, 86 F, 1 O
ifa	96	31.59	11.70	11 M, 85 F, 0 O

### Materials

#### Stimuli

As stimuli, participants were presented with summarised definitions of mental health conditions. The mental health conditions were selected from the ICD-10 (World Health Organization, 1992) for being expressible in IFN, IFA, and PFP form in ways which were deemed unambiguous and common parlance (e.g. anxiety can commonly be expressed as ‘a person with anxiety’, but is ambiguous in terms of whether it refers to a mental health condition in the form ‘anxious person’, and has no commonly used noun form label). Definitions for the mental health conditions were taken from a medical encyclopaedia and shortened for the purposes of the study (Curry, 2008; Dinsmoor, 2008; Frey & Wadehra, 2008; Frey & Schonbeck, 2008). The sentence structures and the condition labels used can be seen in Table 3.

**Table 3:** Sentence Structure and Mental Health Condition Labels for each Linguistic Form

LINGUISTIC FORM	Structure	Terms
pfp	A person with [term]	schizophrenia/alcoholism/anorexia/agoraphobia
ifa	A(n) [term] person	schizophrenic/alcoholic/anorexic/agoraphobic
ifn	A(n) [term]	schizophrenic/alcoholic/anorexic/agoraphobe]

### Scales

#### Measure of Desire for Social Distance

Desire for social distance was measured using Norman, Windell, & Machanda’s (2012) adaption of the original Bogardus scale (Bogardus, 1925). The adaption sought to assess undergraduate reactions to individuals with mental health illnesses, and showed acceptable reliability (α = 0.93). The scale calls for respondents to rate their willingness to interact with a hypothetical individual. Ratings are given along a 5-point scale (from ‘I certainly would’ [scored 1] to ‘I certainly would not’ [scored 5]) in response to statements. Statements vary in level of intimacy. For example, participants were asked to rate their willingness to ‘have lunch with [the person]’ and in another item to ‘trust [the person] to look after their child’. The average of a participant’s ratings is taken, with a higher average indicating greater desire for social distance. The stimuli, as presented in Table 3, were incorporated in the scale in place of references to a target individual as appeared in the scale’s original study (Norman et al., 2012). Minor adaptations to language in the scale were made to fit the context.

Cronbach’s alpha showed the scale to reach acceptable reliability for all mental health conditions considered in the final analysis. These included schizophrenia (α = 0.94), alcoholism (α = 0.92), agoraphobia (α = 0.93), and anorexia (α = 0.92). Acceptability was judged by the recommended minimum of 0.7 set by Nunnally & Bernstein (1994).

#### Familiarity Measure

Two separate items were devised by the authors for recording familiarity. In order to establish whether the participant has any pre-existing knowledge of the label, the first item asks the respondent to ‘select any mental health conditions [they] were aware of prior to this study’. Because people with experience of the condition (directly or indirectly) may have stronger pre-existing beliefs associated with the label, a second item asks the respondent to ‘select any mental health conditions which someone [they] know, or [they themselves], have experienced’. Each item calls for a selection from a list of the four mental health conditions.

## Procedure

### Overview

Ethical Approval was granted for the study by the University involved in the research. Participation was remote and completed online at the participants’ convenience. No personal contact was made with participants prior to or during the experiment. No time restrictions were in place for completion of the online survey. Participants were advised to expect completion to take approximately 15 min.

### Experiment

At the start of the experiment each participant was unknowingly randomly assigned to one linguistic form which would be used in each of the scales throughout the rest of the experiment.

Participants were first presented with an identical definition of a mental health condition. This was done to ensure a basic knowledge of the condition to inform responses. Participants were then asked to respond to the social distance scale regarding a hypothetical individual with the mental health condition described in their assigned linguistic form (e.g. where assigned PFP form and responding regarding schizophrenia, the participant will have been asked to fill out the scale regarding ‘A person with schizophrenia’). This was repeated for each of the four mental health condition definitions presented – each was followed by a scale using the defined term. The mental health conditions were presented in a fixed order: schizophrenia, alcoholism, agoraphobia, and then anorexia. After they had completed the social distance scale for all of the four mental health conditions, participants were asked which they were familiar with.

## Results

### Preliminary Analysis

#### Removals/Missing data

Little’s (1988) ‘Missing Completely at Random (MCAR)’ test was satisfied for responses regarding schizophrenia ( χ^2^ [94] = 89.41, *p* = .62), alcoholism ( χ^2^ [49] = 59.86, *p* = .14), and anorexia ( χ^2^ [60] = 59.09, *p* = .51), indicating that there was no pattern of selective omission by participants (Little, 1988). As a result, cases with any missing values related to the hypotheses (on the social distance scale and familiarity) were treated by listwise deletion without risk of bias (Kang, 2013). Removed participants, as noted in the ‘Participants’ section, were excluded from all analyses, so the sample described is consistent across the explored mental health conditions.

MCAR was not satisfied for agoraphobia ( χ^2^ [68] = 144.12, *p* < .001) indicating that selective omission of responses risked biasing results, including where treated by deletion. In line with the recommended procedure, data associated with agoraphobia was excluded from primary analysis (Kang, 2013). The data for each mental health condition is tested separately in analysis. Therefore, the results for the remaining three conditions are unaffected by the removal of the data for agoraphobia.

#### Familiarity Responses

The vast majority of respondents reported awareness of each mental health condition prior to taking the survey (i.e. schizophrenia = 283; alcoholism = 289; agoraphobia = 256; anorexia = 288). Due to resulting potential issues with statistical power (Elsayir, 2018), data on prior awareness was excluded from analysis.

#### Demographic Differences Between Complete and Incomplete Responses

A chi-square analysis revealed that there was a significant difference between the gender of respondents who did and did not provide complete and therefore usable data. The relation between these variables was significant, X^2^ (2, n = 406) = 14.98, p < .001. Among the 406 participants who reported their gender, male respondents who began the study were significantly more likely to provide usable responses. No other significant demographic differences were found between the respondents who did and did not provide usable responses.

**Table 4:** Cell sizes

	Experienced	Inexperienced
PFP	Schizophrenia = 48 Alcoholism = 71 Anorexia = 53	Schizophrenia = 52 Alcoholism = 29 Anorexia = 47
IFA	Schizophrenia = 36 Alcoholism = 62 Anorexia = 52	Schizophrenia = 60 Alcoholism = 34 Anorexia = 44
IFN	Schizophrenia = 39 Alcoholism = 67 Anorexia = 57	Schizophrenia = 59 Alcoholism = 31 Anorexia = 41

#### Normal Distribution and Homogeneity of Variance

Normality was analysed using the Kolmogorov-Smirnov test. This showed that the means of desire for social distance were not normally distributed for alcoholism (D(294) = 0.065, p = .005) anorexia (D(294) = 0.131, p < .001) or schizophrenia (D(294) = 0.062, p = .008),, however this does not raise concern as literature shows that ANOVA tests are robust against violation of the assumption of normal distribution (Blanca et al., 2017).Homogeneity of variance was examined using Levene’s test. In the social distance data the variances were equal for alcoholism (F(5,288) = 1.15, p = .34), anorexia (F(5,288) = 0.16, p = .98), and schizophrenia (F(5,288) = 0.56, p = .73).

### Primary Analysis

Using SPSS(IBM, 2017) a 3*2 (linguistic form*prior experience with condition) factorial ANOVA was run for each mental health condition. As the present experiment tested the hypotheses repeatedly to address the three separate mental health conditions, a Bonferroni adjustment was made for multiple comparisons (Chen, Feng & Yi, 2017), by multiplying the original α (0.05) by the number of conditions tested, i.e. 3x.05 = 0.015, which we rounded. The new alpha produced by this adjustment was α = 0.02.

#### Schizophrenia

The main effect of linguistic form on desire for social distance was not significant (F[2, 288] = 0.35, *p* = .71). The main effect of experience on desire for social distance was calculated as significant (F[1, 288] = 15.02, *p* = < 0.001) between no experience (M = 2.54, SD = 0.85) and experience (M = 2.15, SD = 0.79). The effect size (*f* = 0.22) exceeded the convention given by Cohen (1988, 285–287) as a small effect (*f* > 0.1) and was less than that given as a medium effect (*f* < 0.25). The interaction effect between linguistic form and experience was not significant (F[ 2, 288] = 0.44, *p* = .64).

#### Alcoholism

The main effect of linguistic form on desire for social distance was not significant (F[2, 288] = 0.64, *p* = .53). The main effect of experience on desire for social distance was calculated as significant (F[1, 288] = 6.49, *p* = .01) between no experience (M = 2.98, SD = 0.83) and experience (M = 2.72, SD = 0.79). The effect size (*f* = 0.14) exceeded the convention given by Cohen (1988, 285–287) as a small effect (*f* > 0.1) and was less than that given as a medium effect (*f* < 0.25). The interaction effect between linguistic form and experience was not significant (F[2, 288] = 0.05, *p* = .95).

#### Anorexia

The main effect of linguistic form on desire for social distance was not significant (F[2, 288] = 0.91, *p* = .40). The main effect of experience on desire for social distance was calculated as significant (F[1, 288] = 8.94, *p* = .003) between no experience (M = 1.88, SD = 0.67) and experience (M = 1.65, SD = 0.65). The effect size (*f* = 0.17) exceeded the convention given by Cohen (1988, 285–287) as a small effect (*f* > 0.1) and was less than that given as a medium effect (*f* < 0.25). The interaction effect between linguistic form and experience was not significant (F[2, 288] = 0.41, *p* = .67).

### Power Analyses

In order to determine whether the present sample size was sufficient, power analyses were performed for the main effect of language. Results are shown in Table 5.

**Table 5:** Table showing the effect size of linguistic form, the power achieved in the present analysis, and the required sample to achieve power of 0.80 in future studies

	Effect size (*f*)	Power: (Post hoc)	Required Sample: (A Priori)
Schizophrenia	0.05	0.11	3967
Alcoholism	0.07	0.16	2158
Anorexia	0.08	0.21	1526

Effect sizes were below the convention for a small effect (see Cohen 1988, pp. 285–287), and power to detect such a slight effect was low. If these slight effects exist and are not chance differences between conditions, the number of participants required to detect it are extremely high.

## Discussion

The present study investigated stigmatising impact of PFP, IFN and IFA forms of mental health labels. Results do not support the hypothesis that the language form of a mental health label affects stigmatising desire for social distance from the labelled individual. Findings indicate that those with experience with a mental health condition tend to have less desire for social distance. However, they do not support the hypothesis that an effect of language is altered by experience with the mental health condition.

A large proportion of responses left essential questions incomplete and were therefore unusable. Male respondents were significantly more likely to complete the study once begun. However, the sample was primarily female. The reason for this is unclear, however there may be a gender difference in response to the topic (see Tu & Liau (2007) for a more extensive consideration of this phenomenon).

The recruitment method meant the sample included many psychology students who may be expected to have prior awareness of psychological disorders (Szeto, Luong, & Dobson, 2013). A sample with fewer students may produce a more even distribution between the groups. Study 2 addressed this by recruiting a sample from local communities across the UK.

Power analysis on the main effect of language revealed an extremely small effect size suggesting either absence of an effect, or a very slight effect. If a very slight effect exists, extremely large samples are necessary for detection.

## Study 2

### Method

The method for Study 2 is identical to that of Study 1 with exception of the differences outlined below.

### Participants

Of 463 people who started the study, 230 provided usable data. These were composed of 29 males (12.6%), 193 females (83.9%), 4 people identifying as other genders, and 4 people who declined to answer. Age ranged from 18 to 81 with a mean of 49.55 (SD = 12.92) (based on 229 responses that reported age). Participants’ prior experience with each mental health condition is shown in Table 6 below.

**Table 6:** Number of Participants by Familiarity with Condition

	Inexperienced	ExperienceD
Schizophrenia	126	104
Agoraphobia	133	97
AlcoholISM	51	179

Participants were recruited by convenience sampling method. Participation was open, online, to any person meeting the requirement of being aged 18 years or older. Recruitment was conducted via community social media groups representing a variety of settlements (cities, towns, villages) across all regions of the UK.

As in Study 1, participants were randomly assigned to a linguistic form condition using the automated randomization feature in Qualtrics software (Qualtrics, 2019).

Basic descriptive statistics for each of the groups are shown in Table 6.

**Table 7:** Demographics by Linguistic Form Group

Linguistic form	N	Age Mean	Age SD	Gender (Male/Female/Other)
ifn	84	50.40	12.07	12 M, 70 F, 2 O
pfp	72	49.10	13.38	9 M, 62 F, 0 O
ifa	74	49.05	13.51	8 M, 61 F, 2 O

### Scale Reliability

A reliability analysis was run for use of the ‘Bogardus Social Distance Scale – Adapted’ with the sample for Study 2. Chronbach’s alpha showed the scale to reach acceptable reliability for all mental health conditions considered in the final analysis. These included schizophrenia (α = 0.95), alcoholism (α = 0.92), and agoraphobia (α = 0.93). Acceptability was judged by the recommended minimum of 0.7 set by Nunnally & Bernstein (1994).

## Results

### Preliminary Analysis

#### Removals/Missing data

Little’s (1988) ‘Missing Completely at Random (MCAR)’ test was satisfied for responses regarding schizophrenia ( χ^2^ [88] = 93.27, *p* = .33), alcoholism ( χ^2^ [66] = 51.75, *p* = .90), and agoraphobia ( χ^2^ [44] = 24.19, *p* = .99), meaning that cases with missing values could be addressed by listwise deletion without risk of bias (Kang, 2013).

MCAR was not satisfied for anorexia ( χ^2^ [55] = 144.17, *p = <* 0.001). This outcome indicates that selective omission of responses regarding anorexia risked biasing results, including where treated by deletion. In line with the recommended procedure, data associated with anorexia was excluded from primary analysis (Kang, 2013).

#### Familiarity Responses

As with Study 1, the majority of respondents reported awareness of each mental health condition prior to taking the survey (i.e. schizophrenia = 212; alcoholism = 225; agorophobia = 199; anorexia = 216). Therefore, only data regarding prior experience was used.

#### Demographic Differences Between Complete and Incomplete Responses

No significant demographic differences were found between the respondents who did and did not complete the study.

**Table 8:** Cell sizes

	Experienced	Inexperienced
PFP	Schizophrenia = 36 Alcoholism = 54 Agoraphobia = 35	Schizophrenia = 36 Alcoholism = 18 Agoraphobia = 37
IFA	Schizophrenia = 38 Alcoholism = 57 Agoraphobia = 32	Schizophrenia = 36 Alcoholism = 17 Agoraphobia = 42
IFN	Schizophrenia = 30 Alcoholism = 68 Agoraphobia = 30	Schizophrenia = 54 Alcoholism = 16 Agoraphobia = 54

#### Normal Distribution

Normality was analysed using the Kolmogorov-Smirnov test. This showed that the means of desire for social distance were not normally distributed for alcoholism (D(230) = 0.066, p = .017), agoraphobia (D(230) = 0.146, p < .001), or schizophrenia (D(230) = 0.09, p < .001). However, as noted previously ANOVA tests are robust against violation of the assumption of normal distribution. (Blanca et al., 2017).

Homogeneity of variance was examined using Levene’s test. In the social distance data the variances were equal for alcoholism (F(5,224) = 0.58, p = .72),, and schizophrenia (F(5,224) = 1.62, p = .16), but not for agoraphobia (F(5,224) = 3.18, p = .009).

### Primary analysis

As with Study 1, a Bonferroni adjustment (α = 0.02) was used, with main effects of linguistic forms and interaction effects remaining not significant.

#### Schizophrenia

The main effect of linguistic form on desire for social distance was not significant (F[2, 224] = 0.31, *p* = .73). The main effect of experience on desire for social distance was significant (F[1, 224] = 24.65, *p* = < 0.001) between no experience (M = 2.54, SD = 0.88) and experience (M = 1.97, SD = 0.78). The effect size (*f* =. 32) exceeded the convention given by Cohen (1988, 285–287) as a medium effect (*f* > 0.25) and was less than that given as a large effect (*f* < 0.4). The interaction effect between linguistic form and experience was not significant (F[2, 224] = 0.74, *p* = .48).

#### Alcoholism

The main effect of linguistic form on desire for social distance was not significant (F[2, 224] = 0.001, *p* = .99). The main effect of experience on desire for social distance was significant (F[1, 224] = 5.12, *p* = .03) between no experience (M = 2.81, SD = 0.81) and experience (M = 2.53, SD = 0.75). The effect size (*f* = 0.14) exceeded the convention given by Cohen (1988, 285–287) as a small effect (*f* > 0.1) and was less than that given as a medium effect (*f* < 0.25). The interaction effect between linguistic form and experience was not significant (F[ 2, 224] = 0.02, *p* = .98).

#### Agoraphobia

The main effect of linguistic form on desire for social distance was not significant (F[2, 224] = 1.47, *p* = .23). The main effect of experience on desire for social distance was significant (F[1, 224] = 13.42, *p* = < 0.001) between no experience (M = 1.83, SD = 0.73) and experience (M = 1.50, SD = 0.49). The effect size (*f* = 0.23) exceeded the convention given by Cohen (1988, 285–287) as a small effect (*f* > 0.1) and was less than that given as a medium effect (*f* < 0.25). The interaction effect between linguistic form and experience was not significant (F[2, 224] = 0.25, *p* = .78).

### Power Analyses

Power analyses were performed for the main effect of language. Language in the agoraphobia analysis showed a medium effect size (*f* = 0.39) and a high power of detection (1-β = 1), suggesting that the non-significant result has a low chance of being erroneous. For schizophrenia (*f* = 0.08) and alcoholism (*f* = 0.00), extremely small effect sizes were seen (Cohen 1988, pp. 285–287), and power to detect such a small effect was low (1-β = 0.19 & 1-β = 0.05 respectively). If these slight effects exist and are not chance differences between conditions, the number of participants required to detect it are extremely high (N ≥ 1370 & N ≥ 1,735,183 respectively).

## Discussion

Results for Study 2 were similar to those of Study 1. Our results suggest that language form does not affect desire for social distance nor that experience with the mental health condition impacts responses to linguistic form. Both studies showed that those with experience with a mental health condition tend to have less desire for social distance.

Power analysis regarding main effect of language showed a low chance of an erroneous result for agoraphobia. For schizophrenia and alcoholism, they echoed results seen in Study 1. There is either no effect, or very slight effect with extremely large participant pools needed for detection.

In addition, Little’s MCAR test was satisfied for Agoraphobia in Study 2. Together, the experiments produced useable data sets for each of the four mental health conditions and have shown in all instances that there was no effect of linguistic form on desire for social distance. It should be noted that this was only demonstrated among the academic sample for anorexia and among UK local communities for agoraphobia. In Study 1, it was found that male respondents were significantly more likely to leave responses incomplete. This was not found in Study 2, however the majority of participants were again female.

### General Discussion

It has previously been argued that noun form labels equate a person with their disorder and should be avoided to reduce mental health stigma (Snow, 2007). Previous research suggests noun labels result in essentialism, which in turn are associated with stigmatising attitudes (Howell et al., 2011; Maass et al., 2013; Walton & Banaji, 2004). Contrary to our hypotheses, our results suggest that linguistic form (PFP, IFN, IFA) did not influence stigma, as represented by desire for social distance. There is research evidence to suggest that lower levels of empathy and stigmatising attitudes are associated with greater preference for the use of noun-based labels, e.g., “a schizophrenic” (Howell, Ulan, & Powell, 2014; Krzyzanowski, Howell, & Passmore, 2019). This suggests that stigmatising attitudes influence the use of language, rather than the reverse. However, some research (e.g. Cuttler & Ryckman 2019) has shown an impact of linguistic form. A difference between Cuttler and Ryckman’s (2019) study and ours is that while we operationalised stigma in terms of social distance, Cuttler and Ryckman asked participants to rate how well labels such as “aggressive” and “unlikeable” applied under the conditions of different linguistic forms. We therefore suggest that it is important to consider a variety of outcomes related to stigma. It is important to note that desire for social distance is only one facet of stigma. Besides fear/avoidance, other facets include malevolence, authoritarianism, and perceived unpredictability (Kenny et al., 2018). Future studies could explore the effect of the linguistic forms on other facets. Such research should also consider possible influences on the way stigma is manifested, including demographics of the person with the stigmatising attitude and of the person who is the target of the stigmatising attitude (Reavley & Jorm, 2011; Wolff et al., 1996). Other outcomes may be of practical interest, e.g. willingness to seek support for one’s own mental health condition, associated with self-stigma (Yanos, Lucksted, Drapalski, Roe, & Lysaker, 2015).

While we were unable to test the impact of awareness of mental health conditions we did find that prior experience of mental health conditions (reflecting having a condition or knowing someone with the condition) reduced desire for social distance. This may suggest that facilitating experience with mental health conditions may do more to address stigma than linguistic choices. Prior research suggests that interventions facilitating social contact with individuals with mental health conditions can reduce mental health stigma (e.g. Kohrt et al., 2020; Lanfredi et al., 2019). Some forms of social contact may be particularly beneficial; recent research has suggested that the presence of close ties with valued contacts (Pullen et al., 2022), e.g., friends, characterised by positive experiences (Felix & Lynn, 2022) are associated with lower levels of stigma. Social contact is expected to help disconfirm negative stereotypes (Pullen et al., 2022), but this may depend on the nature of that contact. Manago and Krendl (2022) observe that it can be difficult to create social contact outside of controlled research settings. One possible avenue for exploring naturalistic social contact might be in the workplace, which is particularly pertinent given mental health discrimination at work (Dewa, et al., 2021) and in the labour market (Hipes, Lucas, Phelan, & White, 2016).

Interestingly, Manago and Krendl proposed changing attitudes as a mechanism for increasing social contact. Given the mixed evidence suggesting that attitudes may precede language use (e.g. Howell et al., 2014), and that language may influence attitudes (Cuttler & Ryckman, 2019), it is possible that a cyclical relationship exists. We recommend future longitudinal research as a way of exploring the interrelations between these constructs.

Since Study 1 was completed, a study of stigmatised nationality labels has been published which showed that a single exposure to IFN or IFA forms was enough to produce a difference in desire for social distance (Graf et al., 2020). The negative results of the present studies and the positive results of Graf et al. might be explained by stronger, less easily altered, preconceptions being held about mental health conditions than nationality groups. However, before this is accepted as an explanation, methodological differences between Graf et al.’s and the present studies should also be considered. Future studies may wish to adopt elements of the latter’s methodology to investigate mental health labels to determine whether an effect can be better detected by these means.

### Limitations

The first study recruited from sources which would largely be made up of students, and likely also other academics, and interested public. It is unknown how many belonged to each category. The resulting sample was largely young (M = 33.09, SD = 12.20) and female (87.8%). Study 2 therefore recruited from community social media groups spread across the UK, resulting in a slightly higher and more varied age (M = 49.55, SD = 12.92), and a slightly smaller female majority (83.9%). Neither sample can be said to represent demographics of the general UK population (Park, 2021). Future research may wish to use a stratified approach to obtain a more balanced sample (Parsons, 2017).

Our sample may be critiqued also for its size. Statistical power is important in attempting to replicate the findings of other scholars (Hedges & Schauer, 2021). When interaction effects are introduced to experiments, larger sample sizes are required (Brysbaert, 2019). Our sample studies would have benefited from a larger sample. However, except agoraphobia in Study 2, the effect sizes detected would (a) not have met the threshold of even a “small” effect size, and (b) would have required a sample of thousands to be deemed statistically significant. At present we are unsure why agoraphobia should be an exception, or why agoraphobia risked bias in missing data in Study 1. In addition to considering sample size in future studies, agoraphobia may merit particular attention. One possibility may be that collecting data during the relative height of COVID-19 may have influenced responses to items about individuals with a fear of open or crowded spaces.

The present study focused on comparison between linguistic forms. Four mental health conditions were used as stimuli which were presented in a fixed order to participants with no control for order effects. As the order of presentation may affect responses (see Strack, 1992), it is not advisable to use the present results to compare between mental health conditions.

We did not explore all variants of person first and identity first language. For example, while the currently explored PFP form is commonly given as the primary example when advising use of person-first language (American Psychological Association, 2020; Snow, 2007), suggests the following structures; ‘[name] is a [noun label]’, and ‘people who are [adjective]’. Therefore, while the present results do not support some claims made about broad categories of language, we have not tested all linguistic variants. To further understanding, these should be considered in future research.

Only a small number of participants had no prior awareness of the conditions addressed. Therefore it was not possible to test the effects of awareness, as unbalanced group sizes may impact statistical power (Elsayir, 2018). Future research may address this issue by treating familiarity as a continuous variable rather than dichotomous. In addition to measuring self-reported awareness of mental health conditions, it may be useful to measure mental health literacy, which incorporates knowledge of specific conditions and treatments (Wei, McGrath, Hayden, & Kutcher, 2016).

## Conclusion

In conclusion, neither of the present studies found an effect of linguistic form of mental health labels on stigmatising desire for social distance from those they described. Results stand in contrast with literature which has posited that person-first language can reduce stigma, and that which suggests noun forms can be linked to increased stigma. Furthermore, no interaction was found between linguistic form and prior familiarity with conditions. The present studies contribute to a growing body of literature on a complex and underexplored topic. There is a range of methodological possibilities to be considered for future research, and more facets of stigma and variations of language remain to be explored. We suggest that emphasis be placed on challenging stigmatising attitudes through opportunities for social contact with people experiencing mental health challenges.
